# Benefits of Study Participation for Patients with Advanced Cancer Receiving Radiotherapy: A Prospective Observational Study

**DOI:** 10.1089/pmr.2022.0044

**Published:** 2022-11-03

**Authors:** Asta Bye, Ellen Bjerkeset, Hanne Stensheim, Jon H. Loge, Marianne J. Hjermstad, Pal Klepstad, Ragnhild Habberstad, Stein Kaasa, Nina Aass

**Affiliations:** ^1^ Department of Nursing and Health Promotion, Faculty of Health Sciences, OsloMet - Oslo Metropolitan University, Oslo, Norway.; ^2^ European Palliative Care Research Centre, Department of Oncology, Oslo University Hospital, and Institute of Clinical Medicine, University of Oslo, Oslo, Norway.; ^3^ Regional Advisory Unit for Palliative Care, Department of Oncology, Oslo University Hospital, Oslo, Norway.; ^4^ Department of Research, Cancer Registry of Norway, Oslo, Norway.; ^5^ Institute of Basic Medical Sciences, University of Oslo, Oslo, Norway.; ^6^ Department of Circulation and Medical Imaging, Norwegian University of Science and Technology (NTNU), Trondheim, Norway.; ^7^ Department of Anesthesiology and Intensive Care Medicine, St Olavs Hospital, Trondheim University Hospital, Trondheim, Norway.; ^8^ Cancer Clinic, St. Olavs Hospital, Trondheim University Hospital, Trondheim, Norway.

**Keywords:** advanced cancer, palliative care, palliative radiotherapy, patient reported outcomes, study participation

## Abstract

**Background:**

Patients with advanced cancer and bone metastases may have unmet palliative care (PC) needs that go unnoticed during clinical oncological practice. This observational study describes interventions that were initiated as the patients participated in the Palliative Radiotherapy and Inflammation Study (PRAIS). It was hypothesized that the patients would benefit from study participation due to PC interventions initiated by the study team.

**Methods:**

A retrospective review of patients' electronic records. Patients with advanced cancer and painful bone metastases included in PRAIS were eligible. All patients met with the study team before start of radiotherapy, after completion of Patient Reported Outcome Measures. Interventions initiated by the study team were documented in the patients' electronic records.

**Results:**

A total of 133 patients were reviewed: 63% males, mean (standard deviation [SD]) age 65 (9.6) and mean (SD) Karnofsky performance status (KPS) score 73.2 (9.1). Interventions were initiated in 50% (*n* = 67) of the patients. Changes in opioid management (69%), treatment of constipation (43%), and nausea (24%) and nutritional advice were most frequent (21%). Patients receiving interventions had lower mean KPS (70 vs. 77 *p* < 0.001), shorter survival time after study inclusion (median 28 vs. 57.5 weeks *p* = 0.005) and were more often opioid naïve (12% vs. 39% *p* < 0.001) than those not receiving interventions by the study team.

**Conclusions:**

Patients with advanced cancer and painful bone metastasis benefited from study participation due to multiple PC interventions initiated by the study team. The findings call for a systematic integration of PC in patients with advanced cancer.

**Trial Registration:**

ClinicalTrials.gov NCT02107664.

## Introduction

Participation in clinical research for patients with advanced cancer has been questioned ethically, mainly because it can be demanding and benefits are not certain.^1^ Some authors have argued that since these are vulnerable patients, study participation is both disruptive and inappropriate.^2^ However, when patients with life-threatening disease are asked, the majority reports willingness to participate and that they benefit from research participation.^3,4^ The same applies to their family caregivers.

The reported benefits are often related to the complex health status of these patients and personal gains due to interventions improving or maintaining their functioning and quality of life (QoL).^5^ Patients also tend to emphasize the importance of being given the opportunity of making a useful contribution to other patients despite own limited life expectancy.^6^

The Palliative Radiotherapy and Inflammation Study (PRAIS) was initiated with the aim of identifying predictors of pain response after radiotherapy (RT) in patients with painful bone metastases.^7,8^ Adult patients (≥18 years) with a verified cancer diagnosis about to undergo RT with a palliative intent for painful bone metastases were recruited from seven oncological centres across Europe in a longitudinal observational study and followed for one year or until they died.^8^ In addition to undergoing RT, the study patients completed several Patient Reported Outcome Measures (PROMs) for assessment of pain and other symptoms, level of functioning, psychological distress, and QoL.

The patients included at one of the PRAIS study centers (Oslo University Hospital [OUH]) were met and followed by a physician and a nurse experienced in palliative cancer care.^9^ During the first study consultation, this study team observed that many of the included patients also reported common symptoms other than pain, for example, constipation and nausea. In addition, lack of emotional support and need for home-based services were frequently reported. These observations were taken as an indication of unmet palliative care (PC) needs that had not been managed during recent clinical consultations.^10^

Since the study team consisted of competent health care professionals with long PC experience, they could not ignore the patients' unmet needs for ethical reasons and appropriate interventions were offered. Based on this, it was hypothesized that the included patients benefited from study participation due to initiation of several PC interventions. To explore the hypothesis, the electronic hospital records of all patients included in PRAIS at OUH were retrospectively reviewed. The objective was to describe (a) the number and types of PC interventions that were initiated by the study team at the first study consultation, and (b) characteristics of the patients who received these interventions versus those who did not.

## Methods

### Patients

Overall, 574 patients from seven centers in Europe were enrolled in the PRAIS study (ClinicalTrials.gov registration NCT02107664).^8^ Between January 2015 and December 2017, a sample of 179 patients was included at OUH. Inclusion criteria were an established cancer diagnosis, referral to palliative RT for verified computer tomography/magnetic resonance imaging (CT/MRI) painful bone metastases, age ≥18, and ability to comply with trial procedures. Exclusion criteria were on-going RT, RT administered within the previous four weeks, or pathological fracture in long bones. Further details regarding criteria for participation and RT treatment have been presented elsewhere.^8^

All patients referred to OUH for palliative RT have a routine appointment at the oncology outpatient clinic before the start of CT dose planning and RT. This scheduled appointment with an oncologist consists of a clinical interview addressing the patients' symptoms, standard clinical examination, supplemented with blood tests, additional imaging, and other examinations if necessary. Based on this, the indications for RT are confirmed. The oncologist then plans the RT in detail (total dose and fractionation), informs patients (and their informal caregivers) about the treatment and follow-up plans, and refers them to RT.

These lists of referred patients were screened by the OUH study team for potentially eligible patients for the PRAIS study. The team approached the identified patients when they met for CT dose planning. This resulted in the sample of 179 patients. The present study on unmet needs was confined to outpatients able to travel regularly between their home and the hospital for RT, resulting in a sample of 134 patients.

### Study procedures

The study team evaluated the patients for participation in PRAIS by asking the following two questions, “Do the bone metastases cause you pain?” and “Have you undergone RT the last 4 weeks?” Patients answering “yes” to the first question and “no” to the next were regarded as eligible and received detailed oral information about the PRAIS study. They also received the written study information including the consent form and a set of PROMs. The patients were informed that if they decided to participate, the first study consultation would take place one hour before the first RT fraction, and they were instructed to bring the signed consent form and complete the study questionnaires. The time gap between CT dose planning and start of RT varied from zero to seven days.

The questionnaire packet consisted of the following forms: EORTC QLQ-C15 PAL for health-related QoL,^11^ two questions from the Brief Pain Inventory^12^ on worst and average pain during the past 24 hours, supplemented by two questions about pain at the planned irradiated site at rest and movement, respectively (11-point numeric rating scale),^13^ the Leeds Assessment of Neuropathic Symptoms and Signs,^14^ the Patient-Generated Subjective Global Assessment of nutritional status,^15^ and the Patient Health Questionnaire regarding depression.^16^

As PRAIS was an observational study, no interventions other than RT were planned according to the PRAIS protocol, and the patient responses on the questionnaires (baseline, weeks 3, 8, 16, 24 and 52) were intended for study purposes only. The study team was responsible for the consultations before RT. Here, the primary focus was to obtain the necessary information to complete the case report forms (CRFs). However, when the team checked the completeness of the forms with the patient self-reported symptoms, they often discovered symptoms of high intensity (i.e., scores ≥4 on the 0–10 numerical rating scales) and other patient needs.

Since patient-centered focus is prioritized in PC, it would be unethical to ignore this and to not intervene. Therefore, when high symptom burden or obvious needs related to, for example, physical function, self-care, or home care services were detected, this was discussed with the patients. If necessary, appropriate interventions or referrals to other health care specialists were initiated and documented in the electronic patient records.

### Data collection

Demographic and clinical data for the present study were extracted from the CRFs. The following variables were selected: age, gender, living situation, length of education, primary diagnosis, and date thereof (month, year), metastases to other than skeletal and Karnofsky performance status (KPS) scale.^17^ Primary cancer diagnoses were grouped as: breast, urological, lung, gastro-intestinal (GI), and unknown. Urological cancer included prostate, bladder, and kidney; lung cancer also included mesothelioma and thymoma; and GI cancer included all cancers in the GI tract.

In 2018, when the inclusion of patients for the PRAIS study was completed, a systematic retrospective review of the baseline consultations and the related interventions documented in the patients' electronic records was performed. For this purpose, the study team developed a data extraction tool to ensure consistency in the data extraction.

Experienced clinicians (three oncologists, two nurses) developed the tool in an iterative process, based on clinical judgment and experience. Main areas covered by the extraction tool were medication issues, that is, prescription of drugs and correction of doses, non-pharmacological interventions, and referrals to other health care professionals or services.

### Statistics

Statistical analyses were performed using the software SPSS version 25 (IBM Corp., Armonk, NY). Data are presented with descriptive statistics; categorical variables as frequency with percentages and continuous variables as mean with standard deviation (SD). To compare the characteristics of the patients who got PC interventions with those who did not, Pearson Chi-Square tests were used on categorical variables, and two-tailed *t* tests on continuous variables.

The significance level was set at 5%. Survival time was calculated from date of study inclusion. Date of death was extracted from the electronic patient records, with the last update being June 2022.

### Ethical considerations

The Regional Committee for Medical and Health Research Ethics, Central Norway approved the PRAIS study and the amendment for this sub-study (2013/1126/REK Middle Norway). All patients gave their written informed consent before inclusion. The study was carried out in accordance with International Council for Harmonisation of Technical Requirements for Pharmaceuticals for Human Use Good Clinical Practice and the World Medical Association Declaration of Helsinki (1964).

## Results

Of the 134 included outpatients, one withdrew the consent to participation after the baseline consultation with the study team, leaving a study sample of 133. Patient characteristics are listed in Table 1. The sample consisted of 63% males, mean age was 65 years (SD 9.6), and mean KPS score 73 (SD 9.1). The most common cancer diagnoses were GI (31%), urological (31%), and lung (19%). Seventy-five percent of the patients also had metastases to other sites (in addition to the bone metastases, with 57% having two or more (non-bone metastases). At the last update of death (June 2022), 122 patients (92%) had died. Median survival time after study inclusion was 38.0 (1–372) weeks, and 27 (23.3%) had died within three months from inclusion.

**Table 1.** Patient Characteristics and Comparison between Patients Receiving and Those Not Receiving Clinical Interventions

	Total (***N*** = 133), ***n*** (%)	Interventions (***n*** = 67), ***n*** (%)	No interventions (***n*** = 66), ***n*** (%)	*p* ^d^
Age				
Mean years (SD)	65 (9.6)	65.0 (10.3)	64.8 (9.0)	*0.92*
KPS				
Mean score (SD)	73.2 (9.1)	69.7 (7.8)	76.7 (9.0)	*<0.001*
Gender				
Male	84 (63.2)	39 (58.2)	45 (68.2)	*0.28*
Female	49 (36.8)	28 (41.8)	21 (31.8)	
Living conditions				
Alone	30 (22.6)	18 (26.9)	12 (18.2)	*0.41*
Spouse/partner	72 (54.1)	37 (55.2)	35 (53.0)	
Spouse/partner and children	24 (18.0)	9 (13.4)	15 (22.7)	
Children^a^	7 (5.3)	3 (4.5)	4 (6.1)	
Educational status, years				
≤12	71 (53.4)	35 (52.2)	36 (54.5)	*0.86*
>12	62 (46.6)	32 (47.8)	30 (45.5)	
Type of cancer				
Breast	20 (15.0)	10 (14.9)	10 (15.2)	*0.26*
Urological	41 (30.8)	15 (22.4)	26 (39.4)	
Lung^b^	25 (18.8)	14 (20.9)	11 (16.7)	
Gastro-intestinal	41 (30.8)	25 (37.3)	16 (24.2)	
Unknown origin	6 (4.5)	3 (4.5)	3 (4.5)	
Location of other metastases^c^				
Liver	52 (39.1)	30 (44.8)	22 (33.3)	*0.21*
CNS	5 (3.8)	4 (6.0)	1 (1.5)	*0.37*
Lung	45 (33.8)	28 (41.8)	17 (25.8)	*0.07*
Other	73 (54.9)	38 (56.7)	35 (53.0)	*0.72*
Opioid naïve	34 (25.6)	8 (11.9)	26 (39.4)	*<0.001*

^a^ Children >18 years included.

^b^ Including mesothelioma (*n* = 2) and thymoma (*n* = 1).

^c^ Percentage exceeds 100, due to multiple metastases.

^d^ Significance level *p* < 0.05.

CNS, central nervous system; KPS, Karnofsky performance status; SD, standard deviation.

The PC interventions were initiated in 50% (*n* = 67) of the patients at the discretion of the PRAIS study team at the baseline study consultation. The highest proportion of interventions was performed in patients with GI cancers (37%). Patients who received PC interventions had a significantly lower mean KPS (70 vs. 77, *p* < 0.001) and a shorter survival time after study inclusion (median 28 vs. 57.5 weeks, *p* = 0.005) compared with those who did not receive any interventions.

Number and types of clinical interventions initiated by the study team are listed in Table 2. Of the 99 (74%) patients who already received opioids at inclusion, 20 needed dose adjustments and 12 needed advice on how to manage their previously prescribed opioids. Three out of 34 opioid naive patients were started on opioids at study inclusion. When including these patients, totally 46 patients received interventions related to opioid management (start of opioids, switch, adjustment and advices on self-management, especially extra doses when needed). For 16 patients, opioid management was the only intervention (adjustment of dose 13 patients, start of opioids 3 patients).

**Table 2.** Numbers and Types of Clinical Interventions Initiated by the Palliative Radiotherapy and Inflammation Study Team

Interventions	***n*** (%)
No. of patients receiving opioid management (*n* = 46)	
Started naive	3 (6.5)
Switch	11 (23.9)
Adjustment dose	20 (43.5)
Advice on self-management	12 (26.1)
No. of prescriptions other than opioids^a^ (*n* = 64)	
Non-opioid analgesics	10 (15.6)
Laxantia	29 (45.3)
Antiemetics	16 (25.0)
Other^b^	9 (14.1)
No. of patients referred to other health care services^a^ (*n* = 23)	
Total no. of referrals to other health care services (*n* = 30)	
Specialized palliative care at local hospitals	13 (43.3)
Outpatient departments, OUH^c^	5 (16.7)
Community health care services	4 (13.3)
Other health care professionals, OUH^d^	5 (16.7)
Hospitalization at OUH	3 (10.0)

^a^ Several patients received more than one prescription/referral.

^b^ Gastric ulcer prophylaxis (*n* = 3), antimycotica (*n* = 2), corticosteroids (*n* = 1), benzodiazepines (*n* = 1), discontinuation of medication (*n* = 2), blood transfusion (*n* = 1).

^c^ Radiology (*n* = 2), oncology (*n* = 3).

^d^ Dietitian (*n* = 3), physiotherapist (*n* = 1), priest (*n* = 1).

OUH, Oslo University Hospital.

Most patients receiving palliative interventions received multiple interventions (51 of 67). The consultations revealed that many patients reported relatively high numbers of symptoms, particularly constipation (*n* = 29) and nausea (*n* = 16). A high number of prescriptions were therefore issued (*n* = 43), with laxatives and antiemetic medication being the two most common. General advice on how to handle symptoms, for example, nutrition, oral care, fatigue, sleep disturbances, and whom to contact if the symptoms worsened, were performed in 22 cases.

Psychosocial advice, and issues about activities of daily living were addressed in 13 cases. As shown in Table 2, 23 patients were referred to other health care services. Two patients were referred to radiological examinations due to suspected new bone metastases and deep vein thrombosis, and three to the oncological outpatient clinic for multiple interventions, for example, correction of hypercalcemia, blood transfusions, and problems with a venous access port. For six patients, referrals to multiple services were necessary, and three patients were hospitalized due to severe symptoms.

## Discussion

Findings from this observational study in patients with painful bone metastases indicate that PC needs were insufficiently attended to during the oncological consultations before RT. The patients reported several clinically significant symptoms and complex problems that had not been fully addressed in the ordinary clinical setting. After screening with the PROMs completed by the patients at baseline, the study team initiated interventions for half of them. Patients receiving interventions were characterized by lower KPS and shorter survival time compared with those who did not receive any interventions.

To be able to offer the best possible treatment for all patients, patients with limited life expectancy should also be included in clinical research,^18^ despite the perception of advanced cancer patients being too fragile and often not willing to participate in studies.^6^ The patients enrolled in the present study had a median survival of eight months and about 25% were dead at three months. Even with advanced disease and short survival, most patients agreed to participate in the study when asked and completed repeated questionnaire packets.

Patients with advanced cancer and short survival may, therefore, not be as unwilling to participate in research as many expect.^19^ This observation is in line with former studies showing that patients with life-threatening illness, when asked, are both interested and willing to participate and do not perceive participation as time-consuming or burdensome.^3,18,20^ Patients may choose to continue with the study despite feeling unwell because of a need for information or concerns about care.^3^ Study participation brings on someone to talk to about the concerns.

Others express that it is important to participate in research to be able to help future patients in similar situations.^18^ Study participation has also been identified as beneficial, and thereby of interest to patients due to possibilities of improved symptom control.^19^

Interestingly, the patients who received interventions aiming at improving symptom control in the present study had lower KPS and shorter survival than those who did not have any interventions. This finding is probably due to a more advanced disease in the first group. Based on this, it is highly likely that these patients were representative for those often regarded as too vulnerable to participate in clinical research.^3^ Our study documents that participation may imply direct benefits for the patients regarding symptom control. Several unmet needs were detected, and appropriate interventions were initiated.

However, since this study was not specifically designed as an intervention study, we cannot verify that the interventions led to better symptom control, although this is reasonable to assume, given the nature of the interventions (i.e., opioid adjustments, transfusions, etc.).

All patients in the present study received oncological and/or PC at their local hospitals or in the primary health care. Since it is well known that patients with advanced cancer experience multiple symptoms,^21,22^ symptoms and other palliative needs should ideally be identified and treated by the responsible physicians or care team. One reason for several undetected symptoms in the present population may be new symptoms that had arisen during the time from referral to RT and the meeting with the PRAIS team.

On the other hand, other studies^23,24^ show that it is not uncommon that symptoms such as pain, constipation, nausea, depression, and sleep disturbances go undetected and remain untreated in standard clinical care and therefore are inadequately controlled. A study including patients treated with palliative RT for pain demonstrated that 30% of the patients did not receive any PC the last six months before RT.^24^

These findings underline the premise of the decade-long debate of integration of oncology and PC to improve patient treatment and care.^5^ The fact that half of the patients reported unmet PC needs indicates that they did not receive necessary PC services. Further, a lack of integration of PC into mainstream cancer care was demonstrated, opposed to existing guidelines.^25,26^ Routines for self-report of symptoms by patients that are recognized and managed by health care professionals in a patient-centered approach are crucial for the initiation of care before patients' symptoms severely worsen.^24^ In the present study, the unmet PC needs were disclosed by a short review of the patients' self-reports of symptoms.

Studies have repeatedly shown that routine use of PROMs improves symptom control, perceived QoL, patient and caregiver satisfaction, and communication between clinicians and patients^23,27–29^ and that previously unnoticed problems are disclosed.^30^ Still, barriers toward use of PROMs persist. This may relate to uncertainty about ease and benefits of use, competing demands within established clinical workflows and fear of getting unreadable, missing and faulty data when using paper-based PROMs.^31,32^ Our findings underline the importance of integrating PC into mainstream oncology care as recommended by both the American and European Society of Clinical Oncology^25,26^ based on the positive effects from a series of randomized studies.^23,28,33–36^

In most clinical studies, data collected by PROMs are used as explanatory variables, secondary outcomes, or, less often, as the primary study outcome. Thus, patient responses on PROMs are not evaluated during the studies, by fear of influencing study results. This is not that relevant in an observational study such as PRAIS, particularly so as the effect of RT was defined as the combined measure of pain intensity and opioid dose.

The opioid dose was increased when the patient was considered an RT non-responder even if he or she reported stable or reduced pain. From a clinical point of view, the better the symptom management and health status of the patients, the more likely it is that they comply with treatment over time, achieve the intended effect, and also complete the study.

Study strengths are the long experience in PC by the PRAIS study team, use of PROMs in a systematic way, and use of the data collected by the study team to implement necessary interventions immediately, documentation in the patient records, and communication to follow-up teams. Still some limitations apply. First, the patient population was heterogeneous in terms of disease stage, primary diagnosis, and frailty, which is shown by the range in KPS and survival time from inclusion. Second, the self-report forms were subject to a quick review by the study team, emphasizing on the patient PROMs scores.

However, it may well be that a symptom score of less than 4 also was perceived to be of importance to the individual patient. In addition, people from the study team participated in the development of the data extraction tool after they had consultations with the patients. Thus, it cannot be ruled out that they were aware of their own documentation in the patient records and that this has influenced which data to extract whereas other information may have been overlooked. Given the nature of this study and the fact that this report comes from one of the seven centres in the PRAIS study, results might not be representative of the entire study population.

## Conclusion

More than half of the outpatients included in PRAIS had PC needs at the baseline study consultation, which had not been fully addressed in the ordinary clinical setting. Interventions were related to inadequate analgesic management and treatment of common symptoms such as pain, constipation, and nausea. Patients who needed PC interventions had lower mean KPS and shorter survival time compared with those not in need of such interventions.

Our findings imply a confirmation of our hypothesis that patients with advanced cancer benefit from study participation in terms of getting their unmet needs addressed even in observational, descriptive studies like this. Another take home message is the value of an integration of oncology and PC and systematic use of PROMs as part of patient-centered care.

## Abbreviations Used

CRFs: case report forms
GI: gastro-intestinal
KPS: Karnofsky performance status
OUH: Oslo University Hospital
PC: palliative care
PRAIS: Palliative Radiotherapy and Inflammation Study
PROMs: Patient Reported Outcome Measures
QoL: quality of life
RT: radiotherapy
SD: standard deviation
